# Significance of histological crescent formation in patients with IgA vasculitis (Henoch-Schönlein purpura)-related nephritis: a cohort in the adult Chinese population

**DOI:** 10.1186/s12882-018-1117-9

**Published:** 2018-11-22

**Authors:** Xiao Huang, Jing Wu, Xiao-mei Wu, Ya-xin Hao, Cai-hong Zeng, Zhi-hong Liu, Zheng Tang

**Affiliations:** National Clinical Research Center of Kidney Diseases, Jinling Hospital, Nanjing University School of Medicine, Nanjing, People’s Republic of China

**Keywords:** IgAV-related nephritis, Histological crescent, Therapy, Renal prognosis

## Abstract

**Background:**

IgA vasculitis (IgAV, formerly Henoch-Schönlein purpura) is a type of systemic vasculitis. This study aimed to explore the clinicopathological features, treatment and renal outcomes of adult IgAV-related nephritis (Henoch-Schönlein purpura nephritis) patients with different degrees of crescent formation.

**Methods:**

Adult patients with biopsy-proven IgAV-related nephritis in Nanjing Jinling Hospital were enrolled and divided into three groups as follows: control (no crescents, *n* = 257), group 1 (crescents < 25%, *n* = 381), and group 2 (crescents ≥25%, *n* = 60). The clinicopathological features, treatment and renal outcomes were compared among the three groups.

**Results:**

There were no significant differences in gender and age at biopsy among the three groups. Groups with more crescents had shorter renal durations and higher prevalence of macroscopic hematuria, proteinuria and nephrotic syndrome than the control group. The presence of renal insufficiency at biopsy was similar, whereas laboratory findings indicated that patients with ≥25% crescents had higher levels of serum creatinine and blood urea nitrogen than the control and group 1. Histologically, the incidence of glomeruli-Bowman’s capsule adhesion and capillary necrosis were proportional to the degree of crescent formation. Patients with more crescents received more positive immunosuppressive therapies. During follow-up, the levels of proteinuria and hematuria were in remission after treatment, and patients without crescents had lower levels of proteinuria. At the last follow-up, the renal function had deteriorated in the control and group 1, whereas the levels of serum creatinine at biopsy and last follow-up were similar in group 2. There was a significant difference in renal survival from end-stage renal disease (ESRD) or 50% decline in renal function among the three groups (log-rank, *P* = 0.030). However, no association between crescent formation and renal outcomes was found after adjusting potential confounders.

**Conclusions:**

Adult IgAV-related nephritis patients with more crescents had more-severe renal manifestations and worse treatment responses, whereas the proportions of crescents were not associated with higher risks for ESRD or 50% decline in renal function. A more suitable pathological classification standard is needed to predict renal prognosis.

## Background

Immunoglobulin A (IgA) vasculitis (IgAV), formerly Henoch-Schönlein purpura (HSP), is a systemic small-vessel vasculitis characterized by IgA1-dominant immune deposits. IgAV-related nephritis (Henoch-Schönlein purpura nephritis) is one of the most common causes of secondary glomerulonephritis and is the major cause of mortality in IgAV patients [1–3]. Kidney involvement of IgAV occurs in approximately 30–50% of children [4–6], whereas the incidence of renal involvement with variable outcomes in adults ranges from 45 to 85% of cases [7, 8].

The pathological classification of IgAV-related nephritis in children is carried out according to the International Study of Kidney Disease in Children (ISKDC) pathology grade, which is based in detail on the degree of mesangial proliferation and the presence of crescents [9]. Histological classification is not consensual in adults with IgAV-related nephritis. Pillebout E, et al. have provided a widely accepted classification scheme for IgAV-related nephritis in adults [7]. Some previous studies demonstrated that the degree of crescent formation was a risk factor related to renal prognosis [3, 10, 11]. There was also concern that the presence of crescents was not predictive of the renal outcome [3, 7, 8, 12–14].

The epidemiology, clinical features, and prognosis of IgAV-related nephritis have been well documented [7, 15–18]. In this study, we conducted an analysis of a biopsy-confirmed cohort of patients with IgAV-related nephritis in a Chinese adult population, in order to explore the significance of histological crescent formation. The clinicopathological features, treatment and renal outcomes in adult IgAV-related nephritis patients with different degrees of crescent formation were analyzed.

## Methods

### Patients

Patients with a diagnosis of IgAV-related nephritis confirmed by biopsy [19, 20] from 2003 to 2013 in the Nanjing Jinling Hospital were reviewed. The indication for renal biopsy was the manifestation of hematuria, proteinuria or renal insufficiency in incipient or relapsing patients. Patients aged > 18 years old undergoing renal biopsy were included. Those suffering from diabetes mellitus, chronic liver disease, acute interstitial nephritis, malignancy and other autoimmune disorders were excluded. Patients with follow-up < 12 months were also excluded, in order to minimize unreliability in the estimation of the renal outcome over a short time. The acute and rapidly progressive cases reaching end-stage renal disease (ESRD) within 12 months were included. According to the degree of renal histological crescent formation, the IgAV-related nephritis patients were divided into three subgroups as follows: control (no crescents, *n* = 257), group 1 (crescents < 25%, *n* = 381), group 2 (crescents ≥25%, *n* = 60). All follow-up data were collected until November 2016.

### Clinical and laboratory data at biopsy

Demographic characteristics collected from the medical history such as gender, age at onset and age at biopsy were described. Renal involvement was assessed from the inspection results and manifested as macroscopic hematuria, microscopic hematuria, proteinuria, nephrotic syndrome, or renal insufficiency. Renal duration was defined as the delay between kidney involvement and renal biopsy. Hematuria was measured as erythrocyte counts of  urinary sediment in microscopic examination. Microscopic hematuria was between 10 and 1000 ×10^4^ red cells/mL. Macroscopic hematuria was defined as > 1000 ×10^4^ red cells/mL. Proteinuria was defined as proteinuria > 0.4 g/d. Nephrotic syndrome was characterized as plasma albumin < 35 g/L and proteinuria > 3.5 g/d; patients with hypoalbuminemia < 30 g/L were also included in this category even if the proteinuria was between 3.0 and 3.5 g/d. The estimated glomerular filtration rate (eGFR) was calculated using the chronic kidney disease epidemiology collaboration (CKD-EPI) formula. Renal insufficiency was defined as eGFR < 60 mL/min/1.73 m^2^. Hypertension was defined as blood pressure > 140/90 mmHg or a requirement for anti-hypertensive therapy. The results of the urine tests consisted of baseline hematuria and 24-h urinary protein. Blood indexes including serum creatinine, urea nitrogen, serum uric acid and serum albumin were obtained from routine tests at the time of biopsy.

### Renal pathological data at biopsy

Renal specimens with more than ten glomeruli were considered adequate. Two pathologists, who were unaware of the clinical features, examined the specimens with light microscopy and immunofluorescence independently. Glomerular sclerosis, segmental sclerosis, crescents, glomeruli-Bowman’s capsule adhesion and capillary necrosis were evaluated. The tubulointerstitial lesions, tubular atrophy and interstitial fibrosis were semi-quantitatively graded as none (0), mild (1), moderate (2), or severe (3). Immunofluorescence for immunoglobulin G (IgG), IgA, immunoglobulin M (IgM), complement 3 (C3), and complement 1q (C1q) deposits were semi-quantitatively graded according to the intensity of fluorescence.

### Treatment and renal prognosis

The immunosuppressive therapies, such as methylprednisolone or prednisone, mycophenolate mofetil, tripterysium glycosides and leflunomide, were analyzed after renal biopsy. Renal survival time was calculated from the biopsy to the final follow-up. If patients were lost to follow-up during the study, they were followed until the last recorded visit. The time-average proteinuria (TA-P) was defined as the ratio of the area under the curve of proteinuria during follow-up to the duration of the follow-up [21]. The time-average microscopic hematuria (TA-RBC) and time-average mean arterial pressure (TA-MAP) was calculated with the same method to evaluate the treatment response. The combined end point was ESRD (eGFR < 15 mL/min/1.73 m^2^, initiation of dialysis or transplantation for more than 3 months) or 50% decline in renal function.

### Statistical analysis

Statistical software SPSS 18.0 (SPSS, Chicago, IL, USA) was used for the statistical analysis. Normally distributed variables were expressed as the mean ± SD and analyzed by one-way ANOVA. Multiple comparisons between the groups were performed using the LSD or Tamhane’s T2 method. Non-parametric variables were expressed as median (interquartile range) and compared using either the MannWhitney or Kruskal-Wallis test. Categorical variables were expressed as percentages and compared using a Chi square or Fisher’s exact test. Wilcoxon signed-rank test was used to analyze paired non-parametric data. Pearson or spearman correlation was used to assess the association between two variables. Renal survival was estimated with the Kaplan–Meier method and compared with a log-rank test across groups. The relationship between parameters and renal survival was assessed using Cox regression. All *P*-values were two-tailed and values < 0.05 were considered statistically significant.

## Results

### Demographic characteristics in adults with IgAV-related nephritis

**Fig. 1:**
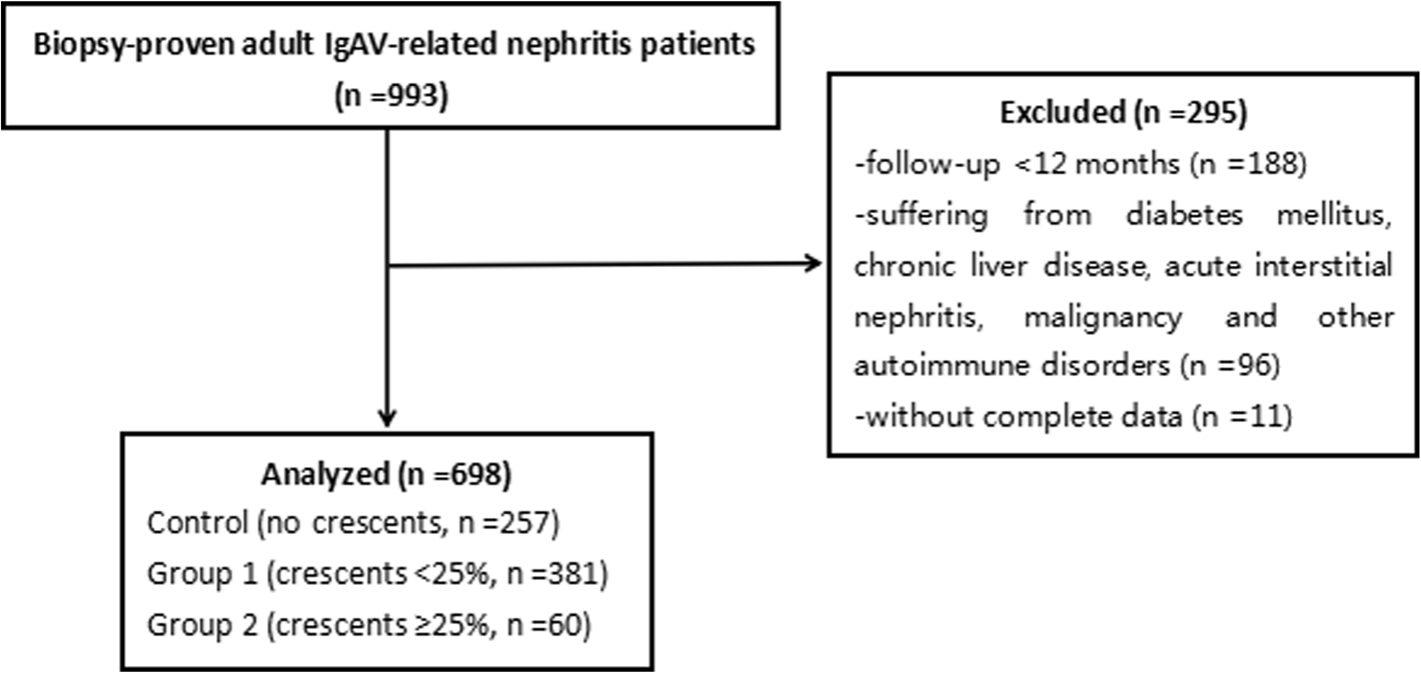
Flow diagram of patient progress

A flow diagram describing the patient samples and exclusions is shown in Fig. 1. The proportions of male patients in the three groups were approximately 54.9, 49.6 and 55.0%, respectively. There were no differences among the three groups. The median ages at biopsy were 30.0 (22.5–39.0), 28.0 (22.0–37.5) and 33.5 (21.0–45.8) years old without significant differences among the groups (*P* = 0.246). The onset age in group 2 was older than that in the control group and group 1. The median renal durations in the control group, group 1, and group 2 were 7.0 (2.0–37.5), 4.0 (1.0–24.0) and 2.0 (1.0–10.8) months, respectively. A negative correlation existed between the renal duration and the degree of crescent formation (Table 1).

**Table 1:** Demographic characteristics at biopsy in adults with IgAV-related nephritis

Parameters	Control (*n* = 257)	Group 1 (*n* = 381)	Group 2 (*n* = 60)	*P*
Male, *n* (%)	141 (54.9)	189 (49.6)	33 (55.0)	*P* = 0.380
Age at biopsy, years	30.0 (22.5–39.0)	28.0 (22.0–37.5)	33.5 (21.0–45.8)	*P* = 0.246
Age at onset, years	26.0 (18.0–36.0)^b^	25.0 (19.0–36.0)^c^	33.5 (20.0–44.8)	*P* = 0.021^*^
Renal duration, months	7.0 (2.0–37.5)^a,b^	4.0 (1.0–24.0)^c^	2.0 (1.0–10.8)	*P* < 0.001^**^

^a^*p* < 0.05 between control and group 1. ^b^ *p* < 0.05 between control and group 2. ^c^ *p* < 0.05 between group 1 and group 2

^**^P < 0.01

### Clinical manifestations at biopsy in adults with IgAV-related nephritis

Patients in group 2 more frequently showed macroscopic hematuria, proteinuria and nephrotic syndrome at biopsy, whereas the incidences of renal insufficiency and hypertension were without significant differences among the three groups. The extra-renal manifestations were also analyzed. The incidences of gastrointestinal symptoms were 25.3, 27.8 and 33.3% in the control group, group 1, and group 2 (*P* = 0.434). Additionally, the presence of arthritis occurred in 28.4, 30.7 and 28.3% of the patients in each group, respectively (*P* = 0.800) (Table 2).

**Table 2:** Clinical manifestations at biopsy in adults with IgAV-related nephritis

Parameters	Control (*n* = 257)	Group 1 (*n* = 381)	Group 2 (*n* = 60)	*P*
Renal involvement				
Macroscopic hematuria, n (%)	10 (3.9)	29 (7.6)	13 (21.7)	*P* < 0.001^**^
Microscopic hematuria, n (%)	185 (72.0)	317 (83.2)	45 (75.0)	*P* = 0.003^**^
Proteinuria, n (%)	184 (71.6)	330 (86.6)	59 (98.3)	*P* < 0.001^**^
Nephrotic syndrome, n (%)	14 (5.4)	34 (8.9)	21 (35.0)	*P* < 0.001^**^
Renal insufficiency, n (%)	15 (5.8)	29 (7.6)	7 (11.7)	*P* = 0.279
Extra-renal manifestation				
Gastrointestinal symptoms, n (%)	65 (25.3)	106 (27.8)	20 (33.3)	*P* = 0.434
Presence of arthritis, n (%)	73 (28.4)	117 (30.7)	17 (28.3)	*P* = 0.800
Hypertension, n (%)	64 (24.9)	68 (17.8)	15 (25.0)	*P* = 0.074

^**^P < 0.01

### Laboratory features at biopsy in adults with IgAV-related nephritis

There were positive correlations between the average levels of both urinary hematuria and protein at renal biopsy and the degree of crescent formation. Although renal insufficiency was not significantly different among the three groups, patients in group 2 had higher levels of serum creatinine and blood urea nitrogen than the control group and group 1. In addition, hemoglobin levels in the group without crescents were significantly higher than those in the groups with crescents (Table 3).

**Table 3:** Laboratory features at biopsy in adults with IgAV-related nephritis

	Control (*n* = 257)	Group 1 (*n* = 381)	Group 2 (*n* = 60)	*P*
Hematuria (10^4^ cells/mL)	56 (10–140)^a,b^	110 (42–258)^c^	282 (109–625)	*P* < 0.001^**^
Urinary protein (g/d)	0.68 (0.35–1.26)^a,b^	0.93 (0.56–1.83)^c^	2.56 (1.36–4.41)	*P* < 0.001^**^
Serum albumin (g/L)	40.5 (37.6–44.2)^a,b^	39.4 (34.9–42.6)^c^	33.6 (28.8–38.6)	*P* < 0.001^**^
Hemoglobin (g/dL)	13.3 ± 1.7^a,b^	12.9 ± 1.7 ^c^	12.3 ± 1.7	*P* < 0.001^**^
Serum creatinine (mg/dL)	0.75 (0.63–0.94)^b^	0.76 (0.63–0.92)^c^	0.96 (0.70–1.06)	*P* = 0.001^**^
Blood urea nitrogen (mg/dL)	12.6 (10.3–16.0)^b^	13.3 (10.6–16.9)^c^	17.6 (13.2–20.6)	*P* < 0.001^**^
Serum uric acid (μmol/L)	344.4 ± 90.7^a^	325.6 ± 85.4	332.7 ± 99.7	*P* = 0.032^*^
Total cholesterol (mmol/L)	4.57 (3.87–5.49)^a,b^	4.78 (4.11–5.79)^c^	5.60 (4.95–7.32)	*P* < 0.001^**^
Hypercholesterolemia, n (%)	45 (17.5)	83 (21.8)	27 (45.0)	*P* < 0.001^**^

^a^*p* < 0.05 between control and group 1. ^b^ *p* < 0.05 between control and group 2. ^c^ *p* < 0.05 between group 1 and group 2

^*^ *P* < 0.05, ^**^ *P* < 0.01

### Renal pathology at biopsy in adults with IgAV-related nephritis

The glomeruli, tubule and interstitial injuries are demonstrated in Table 4. The occurrences of glomeruli-Bowman’s capsule adhesion and capillary necrosis were higher in group 1 and group 2. There were no significant differences in the levels of glomerular sclerosis among all groups. The chronic tubule and interstitial injury, tubular atrophy/interstitial fibrosis, was also similar among all groups. The results of immunofluorescence indicated that the incidence of immunoglobulins and complements in each group were without significant differences.

**Table 4:** Renal pathology at biopsy in adults with IgAV-related nephritis

	Control (*n* = 257)	Group 1 (*n* = 381)	Group 2 (*n* = 60)	*P*
Light microscopy				
Crescents, %	0^a,b^	8.3 (5.0–14.3)^c^	35.6 (29.6–42.0)	*P* < 0.001^**^
Glomeruli-bowman’s capsule adhesion, n (%)	92 (35.8)	206 (54.1)	33 (55.0)	*P* < 0.001^**^
Capillary necrosis, n (%)	29 (11.3)	105 (27.6)	32 (53.3)	*P* < 0.001^**^
Glomerular sclerosis, %	6.3 (0–15.8)	4.5 (0–11.8)	4.9 (0–13.1)	*P* = 0.261
Segmental sclerosis, %	0 (0–7.4)^b^	0 (0–7.4)^c^	0 (0–5.1)	*P* = 0.101
Tubular atrophy/ interstitial fibrosis				
None (0), n (%)	139 (54.1)	230 (60.4)	30 (50.0)	*P* = 0.183
Mild (1), n (%)	92 (35.8)	106 (27.8)	19 (31.7)	
Moderate (2), n (%)	25 (9.7)	42 (11.0)	11 (18.3)	
Severe (3), n (%)	1 (0.4)	3 (0.8)	0 (0)	
Immunofluorescence				
IgG, n (%)	41 (16.0)	63 (16.5)	5 (8.3)	*P* = 0.262
IgM, n (%)	93 (36.2)	139 (36.5)	20 (33.3)	*P* = 0.894
C3, n (%)	220 (85.6)	331 (86.9)	56 (93.3)	*P* = 0.277
C1q, n (%)	6 (2.3)	7 (1.8)	3 (5.0)	*P* = 0.314
IgA and IgG and C3, n (%)	40 (15.6)	61 (16.0)	5 (8.3)	*P* = 0.299

IgA immunoglobulin A; IgG, immunoglobulin G; IgM, immunoglobulin M; C3, complement 3; C1q complement 1q

^a^*p* < 0.05 between control and group 1. ^b^ *p* < 0.05 between control and group 2. ^c^ *p* < 0.05 between group 1 and group 2

^**^P < 0.01

### Correlation between clinical, laboratory parameters and crescents

Correlation analyses showed that renal duration (*r* = − 0.162, *P* < 0.001), hemoglobin (*r* = − 0.197, *P* < 0.001) and serum albumin (*r* = − 0.311, *P* < 0.001) were negatively correlated with renal crescents. In contrast, crescent formation was positively correlated with age at onset (*r* = 0.091, *P* = 0.016), hematuria amount (*r* = 0.326, *P* < 0.001), 24 h urinary protein (*r* = 0.367, *P* < 0.001) and blood urea nitrogen (*r* = 0.147, *P* < 0.001). The other renal lesions, glomeruli-Bowman’s capsule adhesion (*r* = 0.175, *P* < 0.001) and capillary necrosis (*r* = 0.249, *P* < 0.001), were also positively correlated with crescents (Table 5).

**Table 5:** Correlation between clinicopathological data and crescents in adults with IgAV-related nephritis

Parameters	Renal histological Crescent	
	r	*P*
Age at onset	0.091	*P* = 0.016*
Renal duration	−0.162	*P* < 0.001**
Hematuria	0.326	*P* < 0.001**
24 h urinary protein	0.367	*P* < 0.001**
Hemoglobin	−0.197	*P* < 0.001**
Serum creatinine	0.047	*P* = 0.211
Blood urea nitrogen	0.147	*P* < 0.001**
Serum albumin	−0.311	*P* < 0.001**
Glomeruli-Bowman’s capsule adhesion	0.175	*P* < 0.001**
Capillary necrosis	0.249	*P* < 0.001**

* *P* < 0.05, ** *P* < 0.01

### Treatment responses in adults with IgAV-related nephritis

The therapies are listed in Table 6. The proportions of patients treated with methylprednisolone pulse therapy immediately after biopsy were 4.3, 28.1 and 80.0% in the three groups. Of the oral immunosuppressive treatment, the choice of methylprednisolone/prednisone was common among all groups and accounted for 51.4, 75.9 and 88.3% of participants. Corticosteroids were always used with other immunosuppressive agents. The combination of corticosteroids and mycophenolate mofetil represented 2.7, 15.5 and 53.3% of patients in the three groups. Treatment with tripterygium glycosides was also effective and was frequently used in patients without crescents.

**Table 6:** Treatment responses and outcomes in adults with IgAV-related nephritis

	Control (*n* = 257)	Group 1 (*n* = 381)	Group 2 (*n* = 60)	P
Treatment				
methylprednisolone pulse treatment, n (%)	11 (4.3)	107 (28.1)	48 (80.0)	*P* < 0.001^**^
Prednisone/methylprednisolone, n (%)	132 (51.4)	289 (75.9)	53 (88.3)	*P* < 0.001^**^
prednisone + tripterysium glycosides, n (%)	89 (34.6)	174 (45.7)	9 (15.0)	*P* < 0.001^**^
prednisone + leflunomide, n (%)	2 (0.8)	12 (3.1)	0 (0)	*P* = 0.057
prednisone + mycophenolate mofetil, n (%)	7 (2.7)	59 (15.5)	32 (53.3)	*P* < 0.001^**^
Follow-up				
Follow-up time	55.0 (33.0–74.5)	55.0 (35.0–80.0)	51.5 (34.0–81.2)	*P* = 0.813
TA-P (g/d)	0.41 (0.29–0.72)^b^	0.45 (0.29–0.73)^c^	0.56 (0.40–0.97)	*P* = 0.003^**^
< 0.4 g/d	122 (47.5)	158 (41.5)	15 (25.0)	*P* = 0.006^**^
0.4–1.0 g/d	99 (38.5)	160 (42.0)	31 (51.7)	*P* = 0.171
> 1.0 g/d	36 (14.0)	63 (16.5)	14 (23.3)	*P* = 0.203
TA-RBC (10^4^ cells/mL)	27 (10–64)^a,b^	37 (16–91)	58 (20–118)	*P* = 0.001^**^
TA-MAP (mmHg)	92.3 ± 9.8	91.1 ± 8.6	91.6 ± 8.6	*P* = 0.240
Outcome				
Serum creatinine at last follow-up (mg/dL)	0.83 (0.68–1.04)^b^	0.82 (0.67–1.06)^c^	0.98 (0.72–1.12)	*P* = 0.088
50% decline in renal function, n (%)	11 (4.3)	31 (8.1)	8 (13.3)	*P* = 0.027^*^
ESRD, n (%)	7 (2.7)	20 (5.2)	5 (8.3)	*P* = 0.114
ESRD or 50% decline in renal function, n (%)	12 (4.7)	33 (8.7)	8 (13.3)	*P* = 0.037^*^

*TA-P* time-average proteinuria, *TA-RBC* time-average microscopic hematuria, *TA-MAP* time-average mean arterial pressure, *ESRD* end stage renal disease

^a^*p* < 0.05 between control and group 1. ^b^ *p* < 0.05 between control and group 2. ^c^ *p* < 0.05 between group 1 and group 2

^*^ *P* < 0.05, ^**^ *P* < 0.01

**Fig. 2:**
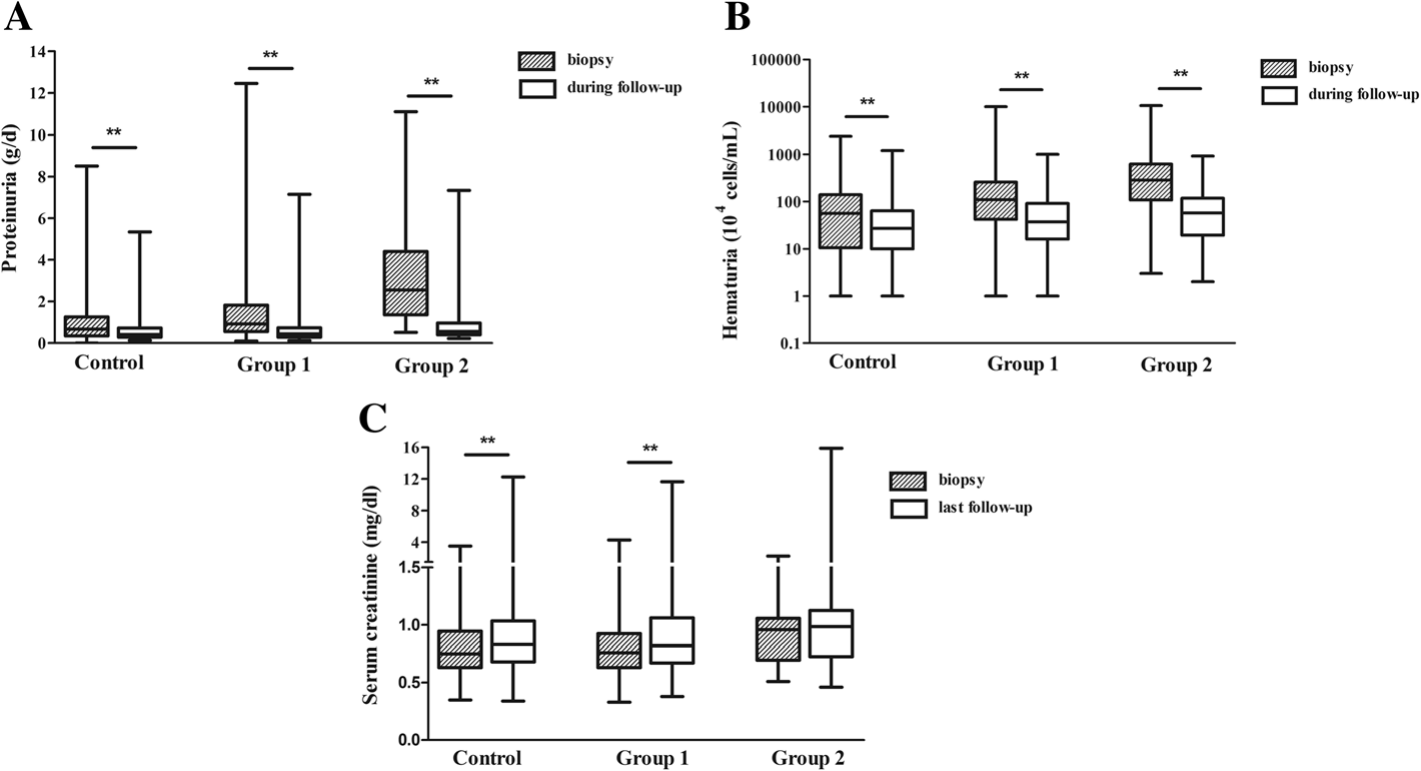
Clinical data at biopsy, during follow-up and at final follow-up in patients with different percentages of crescents. **a** Level of proteinuria at biopsy and during follow-up in the three groups. **b** Level of hematuria at biopsy and during follow-up in the three groups. **c** Level of serum creatinine at biopsy and at final follow up in the three groups. ^**^ *P* < 0.01

During the median follow-up times of 55.0 (33.0–74.5), 55.0 (35.0–80.0) and 51.5 (34.0–81.2) months in the three groups, 47.5, 41.5 and 25.0% of the patients in each group, respectively, reached TA-*P* < 0.4 g/day. The levels of proteinuria and hematuria were in remission after treatment. However, renal function deteriorated gradually in each group. (Figure 2) The levels of serum creatinine at the final follow-up were higher in group 2 than in the other groups. The percentage of patients in each group reaching ESRD or 50% decline in renal function were 4.7, 8.7 and 13.3%, which was consistent with the degree of crescent formation (*P* = 0.037) (Table 6).

### Renal outcome in adults with IgAV-related nephritis

**Fig. 3:**
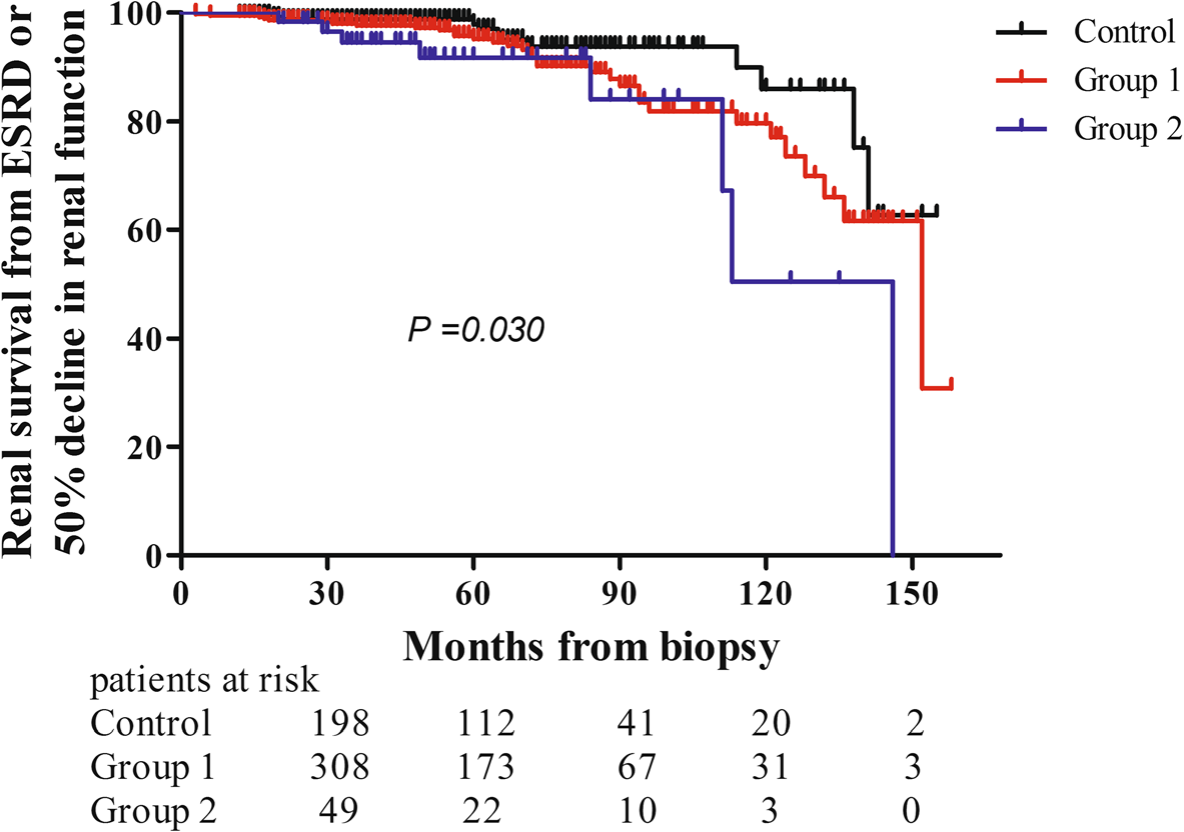
Long-term outcomes of adult IgAV-related nephritis patients in the three groups

Based the Kaplan–Meier methods, the 5-year cumulative rates from ESRD or 50% decline in renal function in the three groups were 97.9% [95% Confidence Interval (CI), 95.7–100%], 95.2% (95% CI, 92.4–97.9%) and 91.7% (95% CI, 83.7–99.7%). The 10-year cumulative rates from the end point among the three groups were 86.0% (95% CI, 74.8–97.2%), 79.7% (95% CI, 71.2–88.1%) and 50.4% (95% CI, 20.0–87.8%). There was no difference in renal prognosis between the control and group 1 (log-rank, *P* = 0.072). The result also turned out that the renal outcome in the control group was poorer than that in group 2 (log-rank, *P* = 0.010). And there was a significant difference in renal survival from ESRD or 50% decline in renal function among the three groups (log-rank, *P* = 0.030). (Figure 3) However, crescent formation failed to reach statistical significance after adjusting for urinary protein > 1.0 g/day, eGFR < 60 mL/min/1.73 m^2^, glomerular sclerosis > 10%, moderate and severe tubular atrophy/ interstitial fibrosis, and immunosuppressive treatment by multivariate Cox regression (Table 7).

**Table 7:** Renal outcome based on categorical grouping of crescent formation by univariate and multivariate Cox regression

Crescent formation	Renal survival from ESRD or 50% decline in renal function			
	Univariate (HR, 95% CI)	P	Multivariate (HR, 95% CI)^a^	P
Control	1.0		1.0	
Group 1	1.798 (0.928–3.485)	0.082	–	0.281
Group 2	3.192 (1.299–7.844)	0.011^*^	–	0.578

^a^Multivariate Cox model: multivariate with urinary protein > 1.0 g/d, eGFR < 60 mL/min/1.73 m^2^, glomerular sclerosis > 10%, moderate and severe tubular atrophy/interstitial fibrosis, and immunosuppressive treatment

^*^P < 0.05

## Discussion

Crescents, as the main evaluation in the pathological classification of ISKDC, might have significance in predicting renal prognosis and guiding therapy [3, 6, 22]. We aimed to define the significance of histological crescent formation in Chinese adult patients with IgAV-related nephritis. The clinical manifestations, pathological parameters, therapy schedules and renal prognosis in adult IgAV-related nephritis patients with different degrees of crescent formation were analyzed in our study.

In previous studies, the majority of patients belonged to ISKDC II and III, and the groups were evenly divided between children and adults. Cases with ISKDC IV and V were limited [8, 9, 23]. In our cohort, only 0.6% of enrolled patients had over 50% crescents in their renal pathological manifestation. In a Korean adult IgAV-related nephritis cohort, crescent formation (ISKDC grade III, IV, and V) was found in 32 (53%) patients, among whom 20 (33%) had crescents involved in ≥50% of glomeruli (grade IV and V) [24]. The duration in the Korean study was shorter than that in our cohort. Furthermore, our results demonstrated a negative correlation between the number of crescents and renal duration. As a result, this variation in the range of crescents in other studies might be attributable to the renal duration. In addition, some patients with specific contraindications, such as anemia caused by severe gastrointestinal hemorrhage, did not undergo kidney biopsy. And some patients might have received positive therapies reversing the active renal lesions before the biopsy, which also influenced the percentage of crescents at biopsy. Therefore, with this limitation of our data, we defined 25% crescents as the cut-off index and divided all patients into three subgroups. Regarding the results of renal pathology in our study, other active lesions, such as glomeruli-Bowman’s capsule adhesion and capillary necrosis, were positively correlated with crescents. A previous study also showed that crescents were frequently seen in association with capillary necrosis and endocapillary proliferation [3].

The extra-renal manifestations, including gastrointestinal symptoms and the presence of arthritis were similar in the three groups. Hypertension, as an independent predictor of renal prognosis, was also without a significant difference between the groups [8, 15]. Acute renal lesions, chronic injuries and antihypertensive treatment were important. In the analysis of renal involvement, this study demonstrated that there were higher proportions of hematuria, proteinuria and nephrotic syndrome in patients with higher percentages of crescents. One study in a population of Chinese children showed that both the 24 h urinary protein contents and urine protein/creatinine levels were higher in patients with ISKDC types IIb, IIIa, and IIIb compared with patients with types I and IIa [9]. Another previous study in adult patients showed that there was no difference in the level of proteinuria between the groups with crescents < 50% and ≥ 50% [24]. This difference might be attributed to different groupings and the sample size.

In the KDIGO (Kidney Disease: Improving Global Outcomes) guideline for adult IgAV-related nephritis, the selection of therapy protocols is mostly based on the clinical features and treatment response, except for the treatment of IgAV-related nephritis patients with crescents in more than 50% of glomeruli in the renal biopsy [6]. The therapy for patients with less than 50% crescents is not clearly stated. With the progressing course of disease, active renal lesions could progress to some chronic pathological manifestations. Previous studies demonstrated that delaying the kidney biopsy could play a role because crescentic glomeruli could rapidly lead to complete glomerulosclerosis if not treated [3, 25]. In addition, in patients with IgA nephropathy, a previous study clearly demonstrated that crescents could be reversed after immunosuppressive treatment, taking advantage of repeat renal biopsy data [26]. The patients in our center with higher degrees of crescent formation received more positive immunosuppressive therapies. Corticosteroid therapy was commonly prescribed in patients, especially in those with a higher percentage of crescents. Corticosteroids combined with other immunosuppressive agents were also used. Previous observational studies supported good outcomes with corticosteroids combined with azathioprine [27], cyclophosphamide [28], cyclosporine [29, 30], plasma exchange [31] and rituximab [32]. However, some other studies did not provide convincing evidence that immunosuppressive therapy, including steroids, had a beneficial effect in patients with IgAV-related nephritis [3, 7, 12, 33–35], and some studies were too small to establish treatment efficacy [30, 36]. As a retrospective study, we simply collected the therapy protocols after the biopsy in each patient and did not conduct a comparison of therapeutic effects with different treatments. Meanwhile, the use of immunosuppressive agents likely resulted in a higher risk of adverse effects, such as infections [37]. Due to the lack of data, our study did not measure the side effects associated with the use of immunosuppressive agents. Large-scale or prospective studies are needed to overcome this limitation.

The results of treatment response found that the proteinuria and hematuria were in remission after treatment. Proteinuria is commonly identified as an independent predictor of renal prognosis, and persistent proteinuria might accelerate the decline of renal function [7]. In our study, more than 50% of patients had abnormal proteinuria during follow-up. The renal function had also deteriorated in two groups at the last follow-up. In addition, more patients without crescents had lower levels of proteinuria and serum creatinine. The Kaplan–Meier analysis showed that the 5- and 10-year cumulative renal survival rates from ESRD or 50% decline in renal function in patients with more crescents was significantly lower. Although the treatment algorithm was more aggressive, the renal prognosis was significantly poorer. A greater awareness of this disease needs to be created among the referring doctors to facilitate early diagnosis and prompt treatment. In the ISKDC classification focusing on mesangial proliferation and the presence of crescents, patients with crescents < 50% were identified as ISKDC III [9], which ignored the dissimilarity with different proportions. In addition, our study showed that crescent formation was not an independent risk factor of renal prognosis after adjusting potential confounders. Previous cohorts demonstrated that tubulointerstitial lesions were strongly related to clinical severity [7, 13, 38]. As a result, a more suitable pathological classification standard is needed to predict renal prognosis and guide therapy. A previous study has suggested that the Oxford classification can be used in predicting long-term outcomes of IgAV-related nephritis [24].

There are some limitations to this study. First of all, data from this study were acquired from a single center. The participants may not have been an adequate representation of the entire Chinese population. Secondly, this was a retrospective study. Patients enrolled were treated with a flexible therapy strategy without an exact duration. In addition, this article focused on the study of crescent in IgAV-related nephritis. The other histological lesions, such as mesangial hypercellularity, endocapillary cellularity and segmental sclerosis, were not analyzed. Therefore, a multicenter study is warranted for the application and evaluation of the Oxford classification of IgAV-related nephritis in adult Chinese patients.

## Conclusions

Adult IgAV-related nephritis patients with more crescents had more-severe renal manifestations and poorer treatment responses. However, the proportions of crescents were not associated with higher risks for end-stage renal disease or 50% decline in renal function after adjusting potential confounders. A more suitable pathological classification standard is needed to predict renal prognosis.

## Abbreviations

C1q: Complement 1q
C3: Complement 3
CI: Confidence interval
CKD-EPI: Chronic kidney disease epidemiology collaboration
eGFR: Estimated glomerular filtration rate
ESRD: End-stage renal disease
HR: Hazard ratio
HSP: Henoch-Schönlein purpura
HSPN: Henoch-Schönlein purpura nephritis
IgA: Immunoglobulin A
IgAV: IgA vasculitis
IgG: Immunoglobulin G
IgM: Immunoglobulin M
ISKDC: International Study of Kidney Disease in Children
TA-MAP: Time-average mean arterial pressure
TA-P: Time-average proteinuria
TA-RBC: Time-average microscopic hematuria

## References

[1] Saulsbury FT (2007). Clinical update: Henoch-Schonlein purpura. Lancet.

[2] Narchi H (2005). Risk of long term renal impairment and duration of follow up recommended for Henoch-Schonlein purpura with normal or minimal urinary findings: a systematic review. Arch Dis Child.

[3] Davin JC (2011). Henoch-Schonlein purpura nephritis: pathophysiology, treatment, and future strategy. Clin J Am Soc Nephrol.

[4] Ghrahani R, Ledika MA, Sapartini G, Setiabudiawan B (2014). Age of onset as a risk factor of renal involvement in Henoch-Schonlein purpura. Asia Pac Allergy.

[5] Chen AC, Lin CL, Shen TC, Li TC, Sung FC, Wei CC (2016). Association between allergic diseases and risks of HSP and HSP nephritis: a population-based study. Pediatr Res.

[6] Kidney Disease: Improving global outcomes (2012). Chapter 11: Henoch-Schonlein purpura nephritis. Kidney Int Suppl (2011).

[7] Pillebout E, Thervet E, Hill G, Alberti C, Vanhille P, Nochy D (2002). Henoch-Schonlein Purpura in adults: outcome and prognostic factors. J Am Soc Nephrol.

[8] Coppo R, Andrulli S, Amore A, Gianoglio B, Conti G, Peruzzi L (2006). Predictors of outcome in Henoch-Schonlein nephritis in children and adults. Am J Kidney Dis.

[9] Ye Q, Shang SQ, Liu AM, Zhang T, Shen HQ, Chen XJ (2015). 24h urinary protein levels and urine protein/creatinine ratios could probably forecast the pathological classification of HSPN. PLoS One.

[10] Edstrom Halling S, Soderberg MP, Berg UB (2010). Predictors of outcome in Henoch-Schonlein nephritis. Pediatr Nephrol.

[11] Shenoy M, Bradbury MG, Lewis MA, Webb NJ (2007). Outcome of Henoch-Schonlein purpura nephritis treated with long-term immunosuppression. Pediatr Nephrol.

[12] Oh HJ, Ahn SV, Yoo DE, Kim SJ, Shin DH, Lee MJ (2012). Clinical outcomes, when matched at presentation, do not vary between adult-onset Henoch-Schonlein purpura nephritis and IgA nephropathy. Kidney Int.

[13] Lu S, Liu D, Xiao J, Yuan W, Wang X, Zhang X (2015). Comparison between adults and children with Henoch-Schonlein purpura nephritis. Pediatr Nephrol.

[14] Jennette JC (2003). Rapidly progressive crescentic glomerulonephritis. Kidney Int.

[15] Shrestha S, Sumingan N, Tan J, Alhous H, McWilliam L, Ballardie F (2006). Henoch Schonlein purpura with nephritis in adults: adverse prognostic indicators in a UK population. QJM.

[16] Rauta V, Tornroth T, Gronhagen-Riska C (2002). Henoch-Schoenlein nephritis in adults-clinical features and outcomes in Finnish patients. Clin Nephrol.

[17] Calvo-Rio V, Loricera J, Mata C, Martin L, Ortiz-Sanjuan F, Alvarez L (2014). Henoch-Schonlein purpura in northern Spain: clinical spectrum of the disease in 417 patients from a single center. Medicine (Baltimore).

[18] Calvino MC, Llorca J, Garcia-Porrua C, Fernandez-Iglesias JL, Rodriguez-Ledo P, Gonzalez-Gay MA (2001). Henoch-Schonlein purpura in children from northwestern Spain: a 20-year epidemiologic and clinical study. Medicine (Baltimore).

[19] Jennette JC, Falk RJ, Bacon PA, Basu N, Cid MC, Ferrario F (2013). 2012 revised international Chapel Hill consensus conference nomenclature of Vasculitides. Arthritis Rheum.

[20] Mills JA, Michel BA, Bloch DA, Calabrese LH, Hunder GG, Arend WP (1990). The American College of Rheumatology 1990 criteria for the classification of Henoch-Schonlein purpura. Arthritis Rheum.

[21] Le W, Liang S, Hu Y, Deng K, Bao H, Zeng C (2012). Long-term renal survival and related risk factors in patients with IgA nephropathy: results from a cohort of 1155 cases in a Chinese adult population. Nephrol Dial Transplant.

[22] Goldstein AR, White RH, Akuse R, Chantler C (1992). Long-term follow-up of childhood Henoch-Schonlein nephritis. Lancet.

[23] Hung SP, Yang YH, Lin YT, Wang LC, Lee JH, Chiang BL (2009). Clinical manifestations and outcomes of Henoch-Schonlein purpura: comparison between adults and children. Pediatr Neonatol.

[24] Kim CH, Lim BJ, Bae YS, Kwon YE, Kim YL, Nam KH (2014). Using the Oxford classification of IgA nephropathy to predict long-term outcomes of Henoch-Schonlein purpura nephritis in adults. Mod Pathol.

[25] Bennett WM, Kincaid-Smith P (1983). Macroscopic hematuria in mesangial IgA nephropathy: correlation with glomerular crescents and renal dysfunction. Kidney Int.

[26] Shen XH, Liang SS, Chen HM, Le WB, Jiang S, Zeng CH (2015). Reversal of active glomerular lesions after immunosuppressive therapy in patients with IgA nephropathy: a repeat-biopsy based observation. J Nephrol.

[27] Foster BJ, Bernard C, Drummond KN, Sharma AK (2000). Effective therapy for severe Henoch-Schonlein purpura nephritis with prednisone and azathioprine: a clinical and histopathologic study. J Pediatr.

[28] Flynn JT, Smoyer WE, Bunchman TE, Kershaw DB, Sedman AB (2001). Treatment of Henoch-Schonlein Purpura glomerulonephritis in children with high-dose corticosteroids plus oral cyclophosphamide. Am J Nephrol.

[29] Shin JI, Park JM, Shin YH, Kim JH, Kim PK, Lee JS (2005). Cyclosporin a therapy for severe Henoch-Schonlein nephritis with nephrotic syndrome. Pediatr Nephrol.

[30] Jauhola O, Ronkainen J, Autio-Harmainen H, Koskimies O, Ala-Houhala M, Arikoski P (2011). Cyclosporine a vs. methylprednisolone for Henoch-Schonlein nephritis: a randomized trial. Pediatr Nephrol.

[31] Kawasaki Y, Suzuki J, Murai M, Takahashi A, Isome M, Nozawa R (2004). Plasmapheresis therapy for rapidly progressive Henoch-Schonlein nephritis. Pediatr Nephrol.

[32] Maritati F, Fenoglio R, Pillebout E, Emmi G, Urban ML, Rocco R (2018). Brief Report: Rituximab for the treatment of adult-onset IgA vasculitis (Henoch-Schönlein purpura). Arthritis Rheumatol..

[33] Pillebout E, Alberti C, Guillevin L, Ouslimani A, Thervet E (2010). Addition of cyclophosphamide to steroids provides no benefit compared with steroids alone in treating adult patients with severe Henoch Schonlein Purpura. Kidney Int.

[34] Ronkainen J, Koskimies O, Ala-Houhala M, Antikainen M, Merenmies J, Rajantie J (2006). Early prednisone therapy in Henoch-Schonlein purpura: a randomized, double-blind, placebo-controlled trial. J Pediatr.

[35] Tarshish P, Bernstein J, Edelmann CM (2004). Henoch-Schonlein purpura nephritis: course of disease and efficacy of cyclophosphamide. Pediatr Nephrol.

[36] Jauhola O, Ronkainen J, Koskimies O, Ala-Houhala M, Arikoski P, Holtta T (2010). Clinical course of extrarenal symptoms in Henoch-Schonlein purpura: a 6-month prospective study. Arch Dis Child.

[37] Lv J, Zhang H, Wong MG, Jardine MJ, Hladunewich M, Jha V (2017). Effect of Oral methylprednisolone on clinical outcomes in patients with IgA nephropathy: the TESTING randomized clinical trial. JAMA.

[38] Coppo R, Mazzucco G, Cagnoli L, Lupo A, Schena FP (1997). Long-term prognosis of Henoch-Schonlein nephritis in adults and children. Italian Group of Renal Immunopathology Collaborative Study on Henoch-Schonlein purpura. Nephrol Dial Transplant.

